# Development of Hydrolysis Probe-Based qPCR Assays for *Panax ginseng* and *Panax quinquefolius* for Detection of Adulteration in Ginseng Herbal Products

**DOI:** 10.3390/foods10112705

**Published:** 2021-11-05

**Authors:** Prasad Kesanakurti, Subramanyam Ragupathy, Adam C. Faller, Dhivya Shanmughanandhan, Francesco Buongiorno, Isabella Della Noce, Zhengfei Lu, Yanjun Zhang, Steven G. Newmaster

**Affiliations:** 1 NHP Research Alliance, College of Biological Sciences, University of Guelph, Guelph, ON N1G 2W1, Canada; ragu@uoguelph.ca (S.R.); afaller@uoguelph.ca (A.C.F.); shanmugd@uoguelph.ca (D.S.); snewmast@uoguelph.ca (S.G.N.); 2 Hyris Ltd., Lower Ground Floor, One George Yard, London EC3V 9DF, UK; francesco.buongiorno@hyris.net (F.B.); isabella.dellanoce@hyris.net (I.D.N.); 3 Herbalife International, Torrance, CA 90502, USA; zhengfeil@herbalife.com (Z.L.); yanjunzh@herbalife.com (Y.Z.)

**Keywords:** panax ginseng, panax quinquefolius, ginseng, herbal medicine, method validation

## Abstract

Authentication of *Panax ginseng* and *Panax quinquefolius* products is important to be able to mitigate instances of adulteration and substitution that exist within the international supply chain of ginseng. To address this issue, species-specific hydrolysis probe qPCR assays were developed and validated for both *P. ginseng* and *P. quinquefolius* herbal dietary supplements. Performance of the probe-based assays was evaluated using analytical validation criteria, which included evaluation of: (1) specificity, in selectively identifying the target species; (2) sensitivity, in detecting the lowest amount of the target material; and (3) repeatability and reproducibility of the method in detecting the target species in raw materials on a real-time PCR platform (reliability). The species-specific probes were developed and successfully passed the validation criteria with 100% specificity, 80–120% efficiency and 100% reliability. The methods developed in this study are fit for purpose, rapid, and easy to implement in quality assurance programs; authentication of ginseng herbal supplements is possible, even with extracts where DNA is fragmented and of low quality and quantity.

## 1. Introduction

Ginseng is the collective term used to refer to several plant species that belong to the genus, *Panax*. Thirteen species of ginseng have been identified, but the most widely used species are *Panax ginseng* (Asian ginseng), grown in China and Korea, and *Panax quinquefolius* (American ginseng), grown in the United States and Canada. Earlier studies have shown that the roots of these two herbs have antidiabetic [1], antistress [2], and anticancer [3] properties. Due to popularity in their use as treatments for diabetes and other diseases, ginseng products are available in different formulations, ranging from whole roots and teas to powders and capsules. In the world medicinal plant trade, ginseng product value is estimated to be greater than USD 2.1 billion [4]. Since ginseng products are valued in their respective domestic markets, intentional adulteration of American ginseng with Asian ginseng and vice versa is commonly practiced, motivated by various economic incentives [5]. Because most ginseng products come in powdered form and have similar morphology, it is difficult to identify the source of these products through visual inspection. Furthermore, these powdered extracts have low quantities of extractable DNA that is broken into small fragments [6,7,8], preventing the use of next generation sequencing (NGS) or conventional DNA barcoding approaches that require long fragments of DNA [9] for species identification.

The most common method used for differentiation of these two *Panax* species involves characterization of metabolites present in the plants. Chemical composition of ginsenosides—the main constituents in *Panax* spp.—varies greatly between these two species; thus, several studies on ginseng adulteration have examined the potential of using ginsenoside composition as a chemical marker [10,11,12]. The movement towards the global trade of botanicals as powdered extract matrices exacerbates the difficulties in ingredient verification due to the lack of chemical extract standards for specific botanicals and their known adulterants. Although there are a few DNA-based studies conducted for detection of adulteration of ginseng, these genetic studies are mainly focused on using DNA markers for studying phylogenetic differences, cultivar identification, and population structure of *Panax* spp. [13,14]. The current methods for detecting adulteration have not been properly validated [15], and they employ older methods such as DNA barcoding, which is not fit for purpose nor effective for the commonly traded highly processed extracts (i.e., the matrices with heavily fragmented DNA template) [6,9]. Several genetic methods have been developed and validated specifically for the identification of ginseng species. However, these methods employed species-specific primers using the conventional PCR approach that involved several post-amplification steps such as gel running and sequencing of the amplified product [16,17,18,19]. Although these methods successfully identified ginseng species, they have the same disadvantages as DNA barcoding. To the authors’ knowledge, there are no validated hydrolysis probe-based qPCR assays available for the sole purpose of identifying adulteration between these two species.

In this study, for the first time, hydrolysis probe-based qPCR assays for *P. ginseng* and *P. quinquefolius* were developed and validated to test the authenticity of labelled *Panax* herbal products. On-site testing facilitates quick and reliable supply chain verification by commercial companies involved in the manufacturing of natural health products. As a novel approach, this assay was validated on a portable qPCR instrument, bCUBE (Hyris Ltd., London, UK), which is a point of care diagnostic device that can be used for on-site testing of botanicals. Since traditional PCR based approaches cannot be used on-site, this approach has the potential to become a handy technique for the botanical manufacturing industry.

## 2. Materials and Methods

### 2.1. Samples for Testing

Both *P. ginseng* and *P. quinquefolius* assays were validated using 38 samples. Nine samples of *P. ginseng* and ten samples of *P. quiquefolius* were used as targets for the respective assays, and the remaining 19 samples were used as non-targets for both assays (Table 1). In addition, 42 labelled, commercial products were procured for *P. ginseng*, and 40 for *P. quinquefolius* from various locations in Canada, China and USA (Table S1) through the study’s partner, the Natural Health Products Research Alliance (NHPRA). Standard biological reference material (SBRM) for *P. ginseng* (BRM661) was obtained from Tieli, Heilongjiang Province, China, through a NHPRA partner. SBRM for *P. quinquefolius* (BRM903) was collected from Brantford, Ontario, Canada. Two types of matrices, root and leaf, were used for testing both the assays.

### 2.2. Primer and Probe Design

Available full length chloroplast genomes of *P. ginseng*, *P. quinquefolius* and *P. notoginseng* were downloaded from Genbank. Chloroplast genomes were aligned using the Windows online version of MAFFT alignment program (https://bit.ly/3iqdjbm, 3 November 2021). Primer and probes were developed from diagnostic regions identified for each species (Tables S2 and S3). Primers and probes for both *P. ginseng* and *P. quinquefolius* were designed using the PrimerQuest^TM^ tool from Integrated DNA Technologies (IDT, Redwood City, CA, USA). Both assays were ordered from IDT (Redwood City, CA, USA) as PrimeTime™, qPCR probe assays, which contained primers and probe, pooled in a single vial. The primers and probe mix was suspended in 1 mL nuclease-free water to obtain a 10× concentration.

### 2.3. DNA Purification and Quantification

Genomic DNA from target and non-target samples was extracted using Nucleospin^®^ Plant II kit (Macherey–Nagel GmbH & Co. KG, Düren, Germany) to obtain high quality DNA. Extractions were performed according to the manufacturer’s instructions, using 60 mg of each sample. DNA was extracted from the target samples (involving both processed and unprocessed (raw) dried root and leaf powder matrices). Similarly, dried leaf material was used for DNA extraction of non-targets. DNA quantification for both targets and non-targets was performed using the Qubit^TM^ 3.0 Fluorometer (Invitrogen, Carlsbad, CA, USA).

### 2.4. Real-Time PCR

The qPCR reactions for validation of both assays were performed on bCUBE (Hyris Ltd., London, UK), a portable qPCR machine, using the SensiFAST^TM^ Probe No-ROX Kit (Bioline, London, UK). PCR was performed in a final volume of 20 μL including 1 μL of templates, 10 μL of SensiFAST Probe No-ROX mix, 1 μL of 250 nM of primers and 125 nM of probe (*P. ginseng*), or 2 μL of 250 nM of primers and 125 nM of probe (*P. quinquefolius*), and made up to 20 μL with nuclease-free water. DNA concentration was normalised to 2 ng/μL for all the samples tested. Reaction volumes of PCR ingredients are shown below (Table 2).

Both *P. ginseng* and *P. quinquefolius* qPCR assays’ cycling conditions on the bCUBE consisted of initial denaturation for 5 min at 95 °C, followed by 40 amplification cycles with denaturation for 10 s at 95 °C, and an annealing/extension for 20 s at 65 °C.

Authentication of 42 *P. ginseng* and 40 *P. quinquefolius* labelled products was performed on a LightCycler^®^ 480 Instrument (Roche Diagnostics, Rotkreuz, Switzerlandwith 35 amplification cycles for both the assays. For the *P. ginseng* assay, an annealing/extension temperature of 67 °C was used, leaving all other cycling conditions the same as the bCUBE test, while no cycling conditions were changed for the *P. quinquefolius* assay as compared with the bCUBE test. To facilitate the uniform testing of 82 commercial products, a LightCycler^®^ 480 Instrument was used, which had the capacity to test 96 samples on each run, instead of bCUBE which could only test 16 samples on each run.

### 2.5. Analytical Specificity

The specificity of an assay is determined by the set of oligonucleotides’ (i.e., primers and/or probe) reactivity with target and non-target species (which may include closely related species from the same genus or other plant species from different genera). Good specificity involves exclusive amplification of intended targets, and no amplification of non-targets. Specificity of *P. ginseng* and *P. quinquefolius* assays was tested on nine and ten target samples, respectively, along with five non-target species—including possible adulterants, congeneric species, and standard biological reference materials (SBRM) with taxonomic herbarium vouchers (Table 1). A no template control (NTC) was included in all validation tests. Specificity of the hydrolysis probe assays was tested following guidelines for validation of qualitative real-time PCR methods for molecular diagnostic identification of botanicals [15]. The specificity test results were conveyed as a percentage of false positives or negatives; the percentage of false positive or false negative results should be zero (see formulae below):

(1) $$\%\;\mathrm{True}\;\mathrm{Positive}\;\left(\mathrm{TP}\right)=\frac{\mathrm{number}\;\mathrm{of}\;\mathrm{correctly}\;\mathrm{classified}\;\mathrm{known}\;\mathrm{samples}}{\mathrm{Total}\;\mathrm{number}\;\mathrm{of}\;\mathrm{known}\;\mathrm{positive}\;\mathrm{samples}}\times \;100$$

(2) $$\%\;\mathrm{False}\;\mathrm{Positive}\;\left(\mathrm{FP}\right)=\frac{\mathrm{number}\;\mathrm{of}\;\mathrm{misclassified}\;\mathrm{known}\;\mathrm{negative}\;\mathrm{samples}}{\mathrm{Total}\;\mathrm{number}\;\mathrm{of}\;\mathrm{known}\;\mathrm{negative}\;\mathrm{samples}}\times \;100$$

(3) $$\%\;\mathrm{False}\;\mathrm{Negative}\;\left(\mathrm{FN}\right)=\frac{\mathrm{number}\;\mathrm{of}\;\mathrm{misclassified}\;\mathrm{known}\;\mathrm{positive}\;\mathrm{samples}}{\mathrm{Total}\;\mathrm{number}\;\mathrm{of}\;\mathrm{known}\;\mathrm{positive}\;\mathrm{samples}}\times \;100\;$$

### 2.6. Analytical Sensitivity

This parameter measures the upper and lower limits of the target and allows for the determination of the limit of detection (LOD) by the assay. To establish the LOD of both *P. ginseng* and *P. quinquefolius* assays, five 10-fold dilutions of target DNA were tested. Two different starting concentrations and two different sample matrices were tested, each in triplicate, to build the standard curves, according to published assay design guidelines [15]. A reaction efficiency of 80–120% with a correlation coefficient R2 ≥ 0.98 was set as a threshold for each standard curve. All replicates were required to be amplified using the same amount of DNA in all tested dilution series.

### 2.7. Repeatability

This parameter measures the percent agreement of results obtained for replicated samples analysed in the same laboratory, by the same operator, on the same device. Repeatability for both *P. ginseng* and *P. quinquefolius* assays was tested on seven and four target samples, respectively, and three non-target samples in triplicate by the same operator, on the same device, on two different days.

### 2.8. Reproducibility

This parameter measures the percent agreement of results obtained for replicated samples analysed in two laboratories, or by two operators. Reproducibility for both *P. ginseng* and *P. quinquefolius* assays was tested on seven and four target samples, respectively, and three non-target samples in triplicate, by two different operators.

## 3. Results

### 3.1. Analytical Specificity

**Figure 1:**
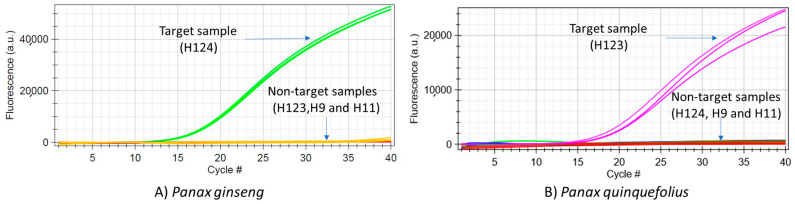
Analytical specificity of *Panax ginseng* assay (**A**), and *Panax quinquefolius* assay (**B**), tested on bCUBE platform shows amplification of only target species. In each run, one target, one no template control and three non-targets were tested in triplicate on a 4 × 4 (16 well) cartridge. All target and non-target samples were tested in different runs using multiple cartridges.

Specificity of the *P. ginseng* assay was assessed using DNA extracts from nine *P. ginseng* and four *P. quiquefolius* samples, and one each of *P. trifolius*, *P. notoginseng*, and the remaining non-targets. Similarly, specificity of the *P. quinquefolius* assay was assessed using DNA extracts from ten *P. quinquefolius* and four *P. ginseng* samples, and one each of *P. trifolius*, *P. notoginseng*, and the remaining non-targets (Table 1). For both assays, all the target samples were amplified, while no amplification curves were noticed for any of the tested non-targets, including the congeneric species *P. notoginseng* and *P. trifolius*. qPCR reactions were performed for 40 cycles on the portable bCUBE platform. Since all the tested target samples were amplified, specificity of the assay, which indicates the ability of assay to correctly detect the target species, was evaluated to be 100% for both assays. Since none of the non-targets were amplified, the percent of false positives, which indicates the ability of the assay to not amplify the non-target species, was zero. Similarly, the percent of false negatives, indicating the incidence of the assay displaying a negative result for a true positive target sample, was also observed to be zero for both assays (Figure 1). Thus, both assays demonstrated superior specificity.

### 3.2. Amplification Efficiency

*Panax ginseng* assay amplification efficiency was assessed to be within the recommended range for all three samples tested. Three samples, PG12, PG56, and H124, with respective DNA concentrations of 10.8, 1.62, and 4.5 ng/uL, were tested. DNA was extracted from processed root powder for samples PG12 and PG56, while DNA was extracted from whole root for sample H124. Each sample was tested in a five serial dilution series, and each dilution was tested with three replicates. All three replicates for each dilution were amplified in all three samples tested. Amplification efficiencies of 84.3%, 85.4%, and 101.2% were recorded for PG12, PG56, and H124, respectively.

**Figure 2:**
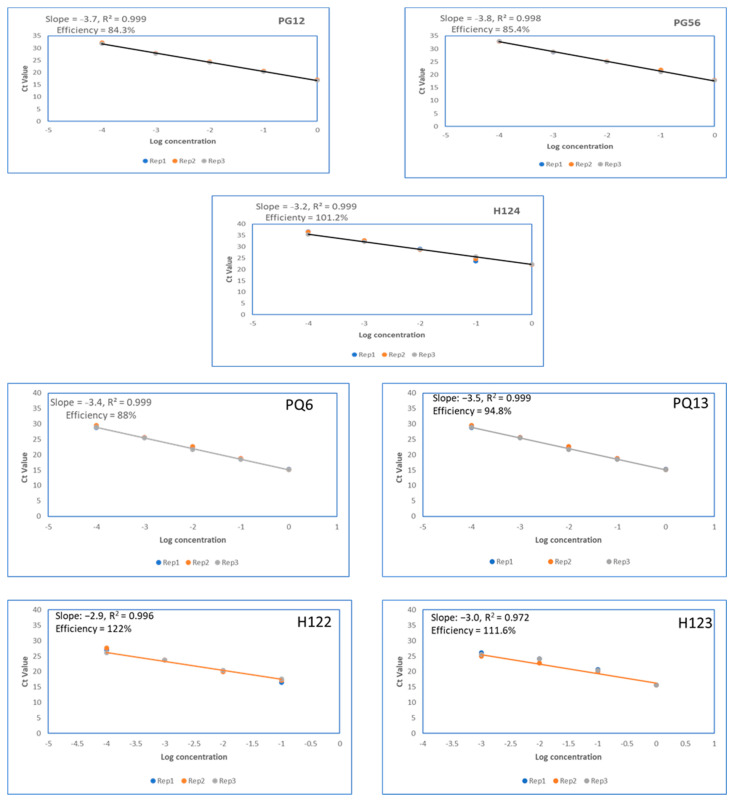
Amplification efficiency of *Panax ginseng* (PG12, PG56, and H124), and *Panax quinquefolius* (PQ6, PQ13, H122, and H123) assays show industrially processed samples (H122, H123, and H124) have efficiency values above 100% and reference samples (PG12, PG56, PQ6, and PQ13) have efficiency values below 100%.

Amplification efficiency of the *P. quinquefolius* assay varied for different sample types and DNA concentrations. Two processed root samples and two reference/raw root samples in powdered form were tested. Each sample was tested in five serial dilution series, and each dilution was tested in three replicates. All three replicates for each dilution were amplified in all four samples tested. Processed samples H122 and H123 were tested at starting DNA concentrations of 27 ng/uL and 33 ng/uL, respectively, and showed amplification efficiencies of 135% and 139%, respectively, when Ct values from all five serial dilutions were included in the analysis. These efficiency values were well above the recommended threshold levels of 80–120%. When Ct values from one of the dilution series were excluded from the analysis, samples H122 and H123 had amplification efficiencies of 122% and 111%, respectively. However, raw root samples PQ6 and PQ13, with Ct values from all five serial dilutions, had amplification efficiencies of 88% (tested with a starting DNA concentration of 4.6 ng/uL) and 94.3% (15.1 ng/uL starting concentration), respectively, (Figure 2).

### 3.3. Analytical Sensitivity

*P. ginseng* assay analytical sensitivity varied among the three tested samples. Samples PG12 and PG56, which had starting DNA concentrations of 10.8 ng/uL and 1.62 ng/uL, had an LOD of 1.08 × 10^−3^ ng and 1.62 × 10^−4^ ng, respectively. However, both samples showed a very similar Ct value of around 32, even with a 10-fold difference in their LOD. Sample H124, which had a starting DNA concentration of 4.5 ng/uL, showed an LOD of 4.5 × 10^−4^ ng, with a Ct value of 36.28.

Analytical sensitivity for the *P. quinquefolius* assay also varied for different sample types. Although processed root samples H122 and H123 had respective DNA concentrations of 27 ng/uL and 33 ng/uL, poor amplification efficiency was noticed when their Ct values crossed 27. As a result, LODs for these samples were determined as 2.7 × 10^−3^ ng and 3.3 × 10^−3^ ng, respectively. Between the samples PQ6 and PQ13, which were dried root samples, PQ6 had a lower DNA concentration of 4.6 ng/uL, and its amplification efficiency was maintained well within the recommended range—even at a Ct value of 35, which corresponded to an LOD of 4.6 × 10^−5^ ng. Similarly, the LOD for sample PQ13 was determined to be 1.51 × 10^−3^ ng.

### 3.4. Reliability

Reliability measures both repeatability and reproducibility of an assay. The *P. ginseng* assay produced very similar Ct values when tested on seven samples of the target *P. ginseng*. None of the tested non-target samples (*P. quinquefolius*) were amplified. Standard deviation (SD) for Ct values obtained on two different dates (repeatability) ranged from 0.03 to 1.05. Similarly, when two different operators tested the assay (reproducibility), SD for Ct values varied from 0.3 to 0.68 (Tables S4 and S5).

The *P. quinquefolius* assay produced very similar Ct values when tested on four samples of the target, *P. quinquefolius*. None of the tested non-target samples (*P. ginseng*) were amplified. Standard deviation (SD) for Ct values obtained on two different dates (repeatability) ranged from 0.04 to 0.3. Similarly, when two different operators tested the assay (reproducibility), SD for target sample Ct values varied from 0.1 to 0.39 (Tables S6 and S7).

### 3.5. Authenticity Testing of Panax Samples

**Figure 3:**
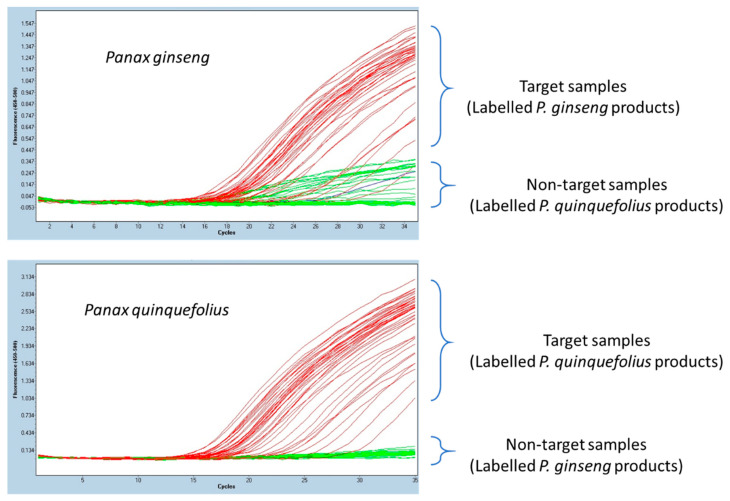
Authentication of commercially labelled ginseng samples with *Panax ginseng* and *Panax quinquefolius* assays using LightCycler^®^ 480 Instrument. A total of 82 samples of known provenance collected from NHPRA members were tested with each assay.

Authenticity testing of ginseng samples, commercially labelled as *P. ginseng* and *P. quinquefolius*, revealed substitution in two cases. Both *P. ginseng* and *P. quinquefolius* assays were tested on 42 samples of *P. ginseng* and 40 samples of *P. quinquefolius*, collected from NHPRA partners. Out of 42 *P. ginseng* samples tested, the *P. ginseng* assay positively identified 40 samples, while two samples 264NAT and 350NAT could not be amplified. None of the 40 *P. quinquefolius* samples were amplified with the *P. ginseng* assay. However, all 40 *P. quinquefolius* samples were positively identified with the *P. quinquefolius* assay, while two samples (264NAT and 350NAT) that were originally labelled as *P. ginseng* were also amplified with this assay, with Ct values of 15.8 and 19.2, respectively. Ct values for both *P. ginseng* and *P. quinquefolius* assays varied from 15 to 30 (Figure 3). The identity of 42 *P. ginseng* and 40 *P. quinquefolius* samples amplified by their respective hydrolysis probe-based assays was confirmed through Sanger sequencing. Two substituted samples, 264NAT and 350NAT, were also confirmed to be P. *quinquefolius* through the Sanger sequencing approach.

## 4. Discussion

There are several studies that have developed tools for DNA-based authentication of ginseng products available in the marketplace. DNA profiling techniques for *Panax* product authentication have been used since the early 1990s. A research group based at The Chinese University of Hong Kong first used random amplified polymorphic DNA (RAPD) and arbitrary primer-based PCR (AP-PCR) for authentication of six *Panax* species and four adulterants [20]. Since then, several DNA-based methods such as RFLP [21] and SCAR [22] have been used for differentiating several *Panax* species. However, all these methods require extensive post-PCR processing, which is labour intensive and time consuming. Additionally, these methods require high quality and quantity DNA, which is difficult to obtain from processed *Panax* herbal products. Similarly, more recently advanced DNA techniques based on next generation sequencing (NGS) have also been used to authenticate *Panax* species. DNA microarray-based technique was used to differentiate *P.* *quinquefolius* from its close relative *P. ginseng* [23]. That study was able to generate 30 polymorphic features specific to *P. ginseng* and nine features specific to *P. quinquefolius* which could be used for further development of SCAR markers for quick identification of *Panax* species. However, this required a very elaborate process that involved various steps such as genomic DNA digestion with two separate restriction enzymes, subtraction of non-target species, cloning of subtracted fragments, and hybridisation of target species with labelled probes. Another NGS study developed a well-supported phylogeny of the genus *Panax* by sequencing full length chloroplast genomes of four *Panax* species and combining them with publicly available chloroplast sequences of the Aralioideae tribe. Although this study was able to differentiate *Panax* species, its main objective was to study the evolutionary relationship of *Panax* group with other members of Araliaceae, rather than specifically developing a diagnostic assay [4]. Additionally, other studies developed DNA-based diagnostic assays for *Panax* species identification. Species-specific primers were developed from indels using a contig based, intron-flanking strategy and successfully differentiated commercial products of *P. ginseng* and *P. quinquefolius* [24]. PCR products were subjected to electrophoresis on 1% agarose gel and the resulting bands were differentiated based on their molecular size. Although this primer-based diagnostic method was successful in *Panax* species identification, it required post-processing of PCR products, which was a multi-step, time consuming process. Similarly, a high resolution melting (HRM) method was developed using plant DNA barcoding markers by designing specific primers for five (ITS2, *matK*, *rbcL*, *trnH-psbA* and *trnL*) barcoding markers [25] and dammarenediol synthase [26]. Although this method did not require any post-PCR processing and measured the real time fluorescence of intercalating dyes, it may not be as reproducible as probe-based techniques. Although these methods were formative, they proved to have limitations, such as only performing well with good quality and quantity DNA template, marked by long sequence reads that are not available in the commonly traded extract forms of ginseng and other botanicals. Hydrolysis probe-based qPCR assays are fit for purpose and efficient, and their >200 bp target size makes the approach robust in the context of highly fragmented DNA matrices.

The first requirement for any diagnostic assay is to have specificity for its target species. Specificity is primarily achieved by targeting species-specific genomic regions for primer and probe design. However, in congeneric species, it can often be difficult to find large stretches of unique regions due to their close taxonomic proximity [27]. In such cases, primers and probes are designed across only one or two single nucleotide polymorphisms (SNPs) [28]. Diagnostic assays targeting SNPs need stringent experimental conditions such as higher annealing temperature and intra-PCR chemical additives to increase efficiency during the assay optimization stage [29]. During this study, it was a challenge to find diagnostic chloroplast regions for both *P. ginseng* and *P. quinquefolius* that could be used to differentiate each from the other, and from other closely related species of the *Panax* genus. Single SNP regions that are unique to *P. ginseng* and *P. quinquefolius* were targeted for probe design. Due to this mononucleotide polymorphism between *Panax* spp., a late amplification of non-targets during assay optimization was noticed; to avoid this, the assay was optimised at a higher annealing temperature and DMSO was added. Cross-reactivity of both assays was tested on most commonly traded botanicals (Table 1) other than *Panax* species, and none of the non-target botanicals were amplified with these assays.

In addition to specificity, other parameters such as amplification efficiency, sensitivity, and reliability are also important for validating a probe-based assay that is designed to identify the ingredients of a herbal product [15]. During the manufacturing process, herbal products undergo several heat and chemical processes that can degrade and reduce the DNA quantity of plant ingredients [7,30,31]. Therefore, it is important for an assay to be effective in identifying ingredients with very low DNA concentrations. In this study, the *P. ginseng* assay had the lowest limit of detection (LOD) of 0.162 picograms of DNA when tested with sample PG56—which is a standard biological reference material (SBRM)—with an average Ct value of 32.89. However, sample H124—which is an industrially processed ginseng root powder—had an LOD of 0.45 picograms, with an average Ct value of 36.28 and with an amplification efficiency of 101.2%. Similarly, the *P. quinquefolius* assay could obtain the lowest limit of detection (LOD), 0.046 picograms, using the sample PQ6 (an SBRM). On the contrary, samples H122 and H123—which are industrially processed root powders—had higher LODs, and their amplification efficiencies surpassed the highest recommended level of 120%, indicating the presence of PCR inhibitory compounds. Although processed samples were observed to impede amplification efficiency in both assays, parameters were observed to remain in range for confident, qualitative ingredient detection. This study clearly demonstrated the ability of both *P. ginseng* and *P. quinquefolius* assays to amplify industrially processed ginseng herbal material.

## Figures and Tables

**Table 1:** Target and non-target species used for validation of *P. ginseng* and *P. quinquefolius* assays.

Botanical Name	Family	Sample Code	Type of Sample	Type of Material
*Panax ginseng*	Araliaceae	PG1	Target	Processed root powder
*Panax ginseng*	Araliaceae	PG3	Target	Dried root powder
*Panax ginseng*	Araliaceae	PG11	Target	Dried root powder
*Panax ginseng*	Araliaceae	PG12/BRM661	Target	Dried root powder (SBRM)
*Panax ginseng*	Araliaceae	PG33	Target	Dried root powder
*Panax ginseng*	Araliaceae	PG27	Target	Dried root powder
*Panax ginseng*	Araliaceae	PG56	Target	Dried root powder
*Panax ginseng*	Araliaceae	PG61	Target	Dried root powder
*Panax ginseng*	Araliaceae	H124	Target	Processed root powder
*Panax quinquefolius*	Araliaceae	H121	Target	Processed root powder
*Panax quinquefolius*	Araliaceae	H122	Target	Processed root powder
*Panax quinquefolius*	Araliaceae	H123	Target	Processed root powder
*Panax quinquefolius*	Araliaceae	PQ6/BRM903	Target	Dried root powder (SBRM)
*Panax quinquefolius*	Araliaceae	PQ7	Target	Dried root powder
*Panax quinquefolius*	Araliaceae	PQ8	Target	Dried root powder
*Panax quinquefolius*	Araliaceae	PQ9	Target	Dried root powder
*Panax quinquefolius*	Araliaceae	PQ13	Target	Dried root powder
*Panax quinquefolius*	Araliaceae	PQ31	Target	Dried root powder
*Panax quinquefolius*	Araliaceae	PQ50	Target	Dried root powder
*Panax notoginseng*	Araliaceae	H125	Non-target	Processed root powder
*Panax trifolius*	Araliaceae	H8	Non-target	Dried root powder
*Glycine max*	Fabaceae	H11	Non-target	Dried leaf powder
*Mirabilis jalapa*	Nyctaginaceae	H126	Non-target	Dried leaf powder
*Phytolacca decandra*	Phytolaccaceae	H9	Non-target	Dried leaf powder
*Curcuma longa*	Zingiberaceae	NV19	Non-target	Dried root powder
*Silybum marianum*	Asteraceae	269NAT	Non-target	Dried seed powder
*Trigonella foenum-graecum*	Fabaceae	CO1002	Non-target	Dried seed powder
*Withania somnifera*	Solanaceae	SKP2	Non-target	Dried root powder
*Camellia sinensis*	Theaceae	281NAT	Non-target	Dried leaf powder
*Valeriana officinalis*	Caprifoliaceae	BT1131GM1258	Non-target	Dried root powder
*Passiflora incarnata*	Passifloraceae	BT1045PR1574	Non-target	Dried leaf powder
*Echinacea purpurea*	Asteraceae	PHY42	Non-target	Dried leaf powder
*Actaea racemosa*	Ranunculaceae	48NBT	Non-target	Dried root powder
*Hydrastis canadensis*	Ranunculaceae	NAT633	Non-target	Dried root powder
*Allium sativum*	Amaryllidaceae	LL0/64-02	Non-target	Dried bulb
*Lepidium meyenii*	Brassicaceae	BT1110RT1285	Non-target	Dried root powder
*Sedum roseum*	Brassicaceae	BT1062AT1020	Non-target	Dried root powder
*Hypericum perforatum*	Clusiaceae	52EU	Non-target	Dried leaf powder

**Table 2:** Ingredients of real-time qPCR used for *P. ginseng* and *P. quinquefolius* assays.

	*Panax ginseng*	*Panax quinquefolius*
SensiFAST Probe No-ROX mix (2×)	10 µL	10 µL
Primer and Probe (10×)	1 µL	2 µL
DMSO	1 µL	1 µL
Template DNA	1 µL	1 µL
Nuclease free water	7 µL	6 µL
Total volume	20 µL	20 µL

## Data Availability

Data is contained within the article or Supplementary Material.

## Supplementary Materials

The following are available online at https://www.mdpi.com/article/10.3390/foods10112705/s1, Table S1. Commercially labelled ginseng products used for authentication testing by both *P. ginseng* and *P. quinquefoius* assays. Table S2. Primer and probe sequences for *P. ginseng* assay. Table S3. Primer and probe sequences for *P. quinquefolius* assay. Table S4. Repeatability of the *P. ginseng* assay. Table S5. Reproducibility of the *P. ginseng* assay. Table S6. Repeatability of the *P. quinquefolius* assay. Table S7. Reproducibility of the *P. quinquefolius* assay. Table S8. Amplicon sequences obtained from *Panax ginseng* commercial products Table S9. Amplicon sequences obtained from *Panax quinquefolius* commercial products Figure S1. *Panax ginseng* amplicon sequence showing variability with *Panax quinquefolius*. Figure S2. *Panax quinquefolius* amplicon sequence showing variability with *Panax ginseng*.
