# Supplementary material for: Development of Hydrolysis Probe-Based qPCR Assays for Panax ginseng and Panax quinquefolius for Detection of Adulteration in Ginseng Herbal Products
Source: Foods. 2021 Nov 5;10(11):2705. doi: 10.3390/foods10112705 (PMC8618564; doi:10.3390/foods10112705)
Supplement: Supplementary file 1 [file foods-10-02705-s001.zip › foods-1418464-supplementary.pdf]

Supplementary Information:

Table S1. Commercially labelled ginseng products used for authentication testing by both *P. ginseng* and *P. quinquefoius* assays.

Sample Code	Species	Product type	Sample Code	Species	Product type
179NAT	*Panax ginseng*	root powder	347NAT	*Panax quinquefolius*	root
180NAT	*Panax ginseng*	root powder	348NAT	*Panax quinquefolius*	root
182NAT	*Panax ginseng*	root powder	249NAT	*Panax quinquefolius*	root
183NAT	*Panax ginseng*	root powder	351NAT	*Panax quinquefolius*	powder
184NAT	*Panax ginseng*	root powder	14NAT	*Panax quinquefolius*	root
201NAT	*Panax ginseng*	root powder	17NAT	*Panax quinquefolius*	root
342NAT	*Panax ginseng*	root powder	339NAT	*Panax quinquefolius*	root
350NAT	*Panax ginseng*	root powder	415NAT	*Panax quinquefolius*	root
401NAT	*Panax ginseng*	root powder	BRM803	*Panax quinquefolius*	root
487NAT	*Panax ginseng*	root powder	112NBT	*Panax quinquefolius*	root
488NAT	*Panax ginseng*	root powder	406NW	*Panax quinquefolius*	powder
3IND	*Panax ginseng*	root powder	1084NW	*Panax quinquefolius*	powder
227BI	*Panax ginseng*	root powder	TNP1	*Panax quinquefolius*	powder
125NAT	*Panax ginseng*	root powder	7PR	*Panax quinquefolius*	powder
236NAT	*Panax ginseng*	leaf	8PR	*Panax quinquefolius*	powder
280NAT	*Panax ginseng*	leaf	24PR	*Panax quinquefolius*	powder
368NAT	*Panax ginseng*	leaf	BRM705	*Panax quinquefolius*	leaf
385NAT	*Panax ginseng*	leaf	BRM871	*Panax quinquefolius*	leaf
431NAT	*Panax ginseng*	leaf	PQ21	*Panax quinquefolius*	leaf
NAT-Nov-2020	*Panax ginseng*	leaf	PQ22	*Panax quinquefolius*	root
JFN1	*Panax ginseng*	root	PQ23	*Panax quinquefolius*	leaf
104JA	*Panax ginseng*	root	PQ24	*Panax quinquefolius*	leaf

106JA	*Panax ginseng*	root	PQ25	*Panax quinquefolius*	root
74NAT	*Panax ginseng*	root	PQ26	*Panax quinquefolius*	leaf
264NAT	*Panax ginseng*	root	PQ27	*Panax quinquefolius*	root
265NAT	*Panax ginseng*	root	PQ28	*Panax quinquefolius*	leaf
PHY1	*Panax ginseng*	root	PQ29	*Panax quinquefolius*	leaf
86NW	*Panax ginseng*	root	PQ30	*Panax quinquefolius*	leaf
287NW	*Panax ginseng*	root	BRM903	*Panax quinquefolius*	root
99NBT	*Panax ginseng*	root	OAC1570	*Panax quinquefolius*	leaf
106NBT	*Panax ginseng*	root	OAC17943	*Panax quinquefolius*	leaf
107NBT	*Panax ginseng*	root	OAC22568	*Panax quinquefolius*	leaf
108NBT	*Panax ginseng*	root	OAC24225	*Panax quinquefolius*	leaf
109NBT	*Panax ginseng*	root	OAC26065	*Panax quinquefolius*	leaf
110NBT	*Panax ginseng*	root	OAC42034	*Panax quinquefolius*	leaf
111NBT	*Panax ginseng*	root	OAC56582	*Panax quinquefolius*	leaf
BRM661	*Panax ginseng*	leaf	OAC56612	*Panax quinquefolius*	leaf
BRM688	*Panax ginseng*	leaf	OAC57071	*Panax quinquefolius*	leaf
BRM689	*Panax ginseng*	leaf	OAC74790	*Panax quinquefolius*	leaf
BRM1039	*Panax ginseng*	leaf	OAC92518	*Panax quinquefolius*	leaf
NAT648	*Panax ginseng*	leaf			
BRM804	*Panax ginseng*	root			

Table S2. Primer and probe sequences for *P. ginseng* assay

Primer FWD	5’- GTCTTGGATAGCCGCGATAAA -3’
Primer REV	5’- GGGACCCTAACCCAAATATTCTT -3’
Probe	5’ - /56-FAM/ CTC ACG CCA /ZEN/ TAT CCG CCA TTC TGA /31AbkFQ/ - 3’

Primer and probes sequences for *P. ginseng* assay were designed from full length chloroplast genome (KM067394). Length of the amplicon for this assay is around 90bp.

Table S3. Primer and probe sequences for *P. quinquefolius* assay

Primer FWD	5’- CCGGTTGCTTTCTGTCCATATAA -3’
Primer REV	5’- AACAGGGTCAGAGGGATCAA -3’
Probe	5’ - /56-FAM/TGC ATA CAG/ZEN/ CTC TAG TTG CCG GTT/31AbkFQ/ - 3’

Primer and probes sequences for *P. quinquefolius* assay were designed from full length chloroplast genome (KT028714). Length of the amplicon for this assay is around 100bp.

Table S4. Repeatability of the *P. ginseng* assay

Sample ID	Sample Type	Species	Date of Analysis	Ct			Ct Mean	SD	CV %
**PG3**	Target	*Panax ginseng*	31/05/2019	20.43	20.66	20.19	20.46	0.03	0.12
			15/06/2019	20.49	20.64	20.32			
**PG11**	Target	*Panax ginseng*	31/05/2019	20.13	20.32	20.11	20.82	0.63	3
			15/06/2019	21.38	21.51	21.44			
**PG33**	Target	*Panax ginseng*	31/05/2019	22.93	23.09	22.23	23.41	0.66	2.82
			15/06/2019	24.12	24	24.08			
**PG61**	Target	*Panax ginseng*	31/05/2019	20.46	20.54	20.08	21.25	0.89	4.19
			15/06/2019	21.48	22.23	22.71			
**PG27**	Target	*Panax ginseng*	31/05/2019	28.86	28.79	27.91	29.57	1.05	3.55
			15/06/2019	29.72	31.19	30.94			
**PG12**	Target	*Panax ginseng*	31/05/2019	20.14	20.39	20.21	20.94	0.69	3.3
			15/06/2019	21.27	21.85	21.76			
**PG56**	Target	*Panax ginseng*	31/05/2019	21.27	21.67	21.33	22.13	0.71	3.19
			15/06/2019	22.51	22.78	23.21			
**PQ13**	Non-target	*Panax quinquefolius*	31/05/2019	N/A	N/A	N/A	N/A	N/A	N/A
			15/06/2019	N/A	N/A	N/A			
**PQ31**	Non-target	*Panax quinquefolius*	31/05/2019	N/A	N/A	N/A	N/A	N/A	N/A
			15/06/2019	N/A	N/A	N/A			
**PQ50**	Non-target	*Panax quinquefolius*	31/05/2019	N/A	N/A	N/A	N/A	N/A	N/A
			15/06/2019	N/A	N/A	N/A			

Table S5. Reproducibility of the *P. ginseng* assay

Sample ID	Sample Type	Species	Date of Analysis	Ct			Ct Mean	SD	CV %
**PG3**	Target	*Panax ginseng*	Operator 1	20.43	20.66	20.19	19.93	0.50	2.51
			Operator 2	20.89	19.35	18.06			
**PG11**	Target	*Panax ginseng*	Operator 1	20.13	20.32	20.11	19.9	0.30	1.48
			Operator 2	19.84	19.63	19.34			
**PG33**	Target	*Panax ginseng*	Operator 1	22.93	23.09	22.23	22.28	0.47	2.11
			Operator 2	22.18	21.91	21.33			
**PG61**	Target	*Panax ginseng*	Operator 1	20.46	20.54	20.08	20.03	0.34	1.67
			Operator 2	20.46	19.74	18.88			
**PG27**	Target	*Panax ginseng*	Operator 1	28.86	28.79	27.91	27.84	0.68	2.44
			Operator 2	28.12	27.17	26.19			
**PG12**	Target	*Panax ginseng*	Operator 1	20.14	20.39	20.21	20.76	0.51	2.46
			Operator 2	21.98	21.36	20.46			
**PG56**	Target	*Panax ginseng*	Operator 1	21.27	21.67	21.33	22.02	0.60	2.72
			Operator 2	23.58	22.74	21.53			
**PQ13**	Non-target	*Panax quinquefolius*	Operator 1	N/A	N/A	N/A	N/A	N/A	N/A
			Operator 2	N/A	N/A	N/A			
**PQ31**	Non-target	*Panax quinquefolius*	Operator 1	N/A	N/A	N/A	N/A	N/A	N/A
			Operator 2	N/A	N/A	N/A			
**PQ50**	Non-target	*Panax quinquefolius*	Operator 1	N/A	N/A	N/A	N/A	N/A	N/A
			Operator 2	N/A	N/A	N/A			

Table S6. Repeatability of the *P. quinquefolius* assay

Sample ID	Sample Type	Species	Date of Analysis	Ct			Ct Mean	SD	CV %
**PQ6**	Target	*Panax quinquefolius*	30/04/2019	19.84	20.67	19.55	20.32	0.30	1.45
			22/05/2019	20.73	20.87	20.22			
**PQ7**	Target	*Panax quinquefolius*	30/04/2019	21.11	20.94	20.81	20.91	0.04	0.19
			22/05/2019	20.86	20.92	20.84			
**PQ50**	Target	*Panax quinquefolius*	30/04/2019	22.28	22.43	22.13	22.38	0.10	0.45
			22/05/2019	22.5	22.49	22.46			
**PQ9**	Target	*Panax quinquefolius*	30/04/2019	21.25	21.55	21.1	21.47	0.17	0.77
			22/05/2019	21.66	21.87	21.35			
**PG3**	Non-target	*Panax ginseng*	30/04/2019	N/A	N/A	N/A	N/A	N/A	N/A
			22/05/2019	N/A	N/A	N/A			
**PG11**	Non-target	*Panax ginseng*	30/04/2019	N/A	N/A	N/A	N/A	N/A	N/A
			22/05/2019	N/A	N/A	N/A			
**PG33**	Non-target	*Panax ginseng*	30/04/2019	N/A	N/A	N/A	N/A	N/A	N/A
			22/05/2019	N/A	N/A	N/A			

Table S7. Reproducibility of the *P. quinquefolius* assay

Sample ID	Sample Type	Species	Operator	Ct			Ct Mean	SD	CV %
**PQ6**	Target	*Panax quinquefolius*	Operator 1	19.84	20.67	19.55	20.41	0.39	1.91
			Operator 2	20.99	21.33	20.08			
**PQ7**	Target	*Panax quinquefolius*	Operator 1	21.11	20.94	20.81	21.23	0.28	1.32
			Operator 2	22.53	21.12	20.89			
**PQ50**	Target	*Panax quinquefolius*	Operator 1	22.28	22.43	22.13	22.5	0.22	0.96
			Operator 2	23.38	22.8	21.94			
**PQ9**	Target	*Panax quinquefolius*	Operator 1	21.25	21.55	21.1	21.4	0.10	0.47
			Operator 2	22.5	20.81	21.19			
**PG3**	Non-target	*Panax ginseng*	Operator 1	N/A	N/A	N/A	N/A	N/A	N/A
			Operator 2	N/A	N/A	N/A			
**PG11**	Non-target	*Panax ginseng*	Operator 1	N/A	N/A	N/A	N/A	N/A	N/A
			Operator 2	N/A	N/A	N/A			
**PG33**	Non-target	*Panax ginseng*	Operator 1	N/A	N/A	N/A	N/A	N/A	N/A
			Operator 2	N/A	N/A	N/A			

Table S8. Amplicon sequences obtained from *Panax ginseng* commercial products

>PHY1 _Pangiprobe
CTCACGCCATATCCGCCATTCTGACCTTTTTCCAGTGAAAGAATATTTGGGTTAGGGTCCC
>108NBT Pangiprobe
CTCACGCCATATCCGCCATTCTGACCTTTTTCCAGTGAAAGAATATTTGGGTTAGGGTCCC
>111NBT Pangiprobe
CTCACGCCATATCCGCCATTCTGACCTTTTTCCAGTGAAAGAATATTTGGGTTAGGGTCCC
>201NAT Pangiprobe
CTCACGCCATATCCGCCATTCTGACCTTTTTCCAGTGAAAGAATATTTGGGTTAGGGTCCC
>227BI Pangiprobe
CTCACGCCATATCCGCCATTCTGACCTTTTTCCAGTGAAAGAATATTTGGGTTAGGGTCCC
>236NAT Pangiprobe
CTCACGCCATATCCGCCATTCTGACCTTTTTCCAGTGAAAGAATATTTGGGTTAGGGTCCC
>249NAT Pangiprobe
CTCACGCCATATCCGCCATTCTGACCTTTTTCCAGTGAAAGAATATTTGGGTTAGGGTCCC
>287NW Pangiprobe
CTCACGCCATATCCGCCATTCTGACCTTTTTCCAGTGAAAGAATATTTGGGTTAGGGTCCC
>348NAT Pangiprobe
CTCACGCCATATCCGCCATTCTGACCTTTTTCCAGTGAAAGAATATTTGGGTTAGGGTCCC
>385NAT Pangiprobe
CTCACGCCATATCCGCCATTCTGACCTTTTTCCAGTGAAAGAATATTTGGGTTAGGGTCCC
>401NAT Pangiprobe
CTCACGCCATATCCGCCATTCTGACCTTTTTCCAGTGAAAGAATATTTGGGTTAGGGTCCC
>431NAT Pangiprobe
CTCACGCCATATCCGCCATTCTGACCTTTTTCCAGTGAAAGAATATTTGGGTTAGGGTCCC
>99NBT Pangiprobe
CTCACGCCATATCCGCCATTCTGACCTTTTTCCAGTGAAAGAATATTTGGGTTAGGGTCCC
>BRM1039 Pangiprobe
CTCACGCCATATCCGCCATTCTGACCTTTTTCCAGTGAAAGAATATTTGGGTTAGGGTCCC
>BRM661 Pangiprobe
CTCACGCCATATCCGCCATTCTGACCTTTTTCCAGTGAAAGAATATTTGGGTTAGGGTCCC
>339NAT Pangiprobe
CTCACGCCATATCCGCCATTCTGACCTTTTTCCAGTGAAAGAATATTTGGGTTAGGGTCCC
>109NBT Pangiprobe
CTCACGCCATATCCGCCATTCTGACCTTTTTCCAGTGAAAGAATATTTGGGTTAGGGTCCC
>110NBT Pangiprobe
CTCACGCCATATCCGCCATTCTGACCTTTTTCCAGTGAAAGAATATTTGGGTTAGGGTCCC
>415NAT Pangiprobe
CTCACGCCATATCCGCCATTCTGACCTTTTTCCAGTGAAAGAATATTTGGGTTAGGGTCCC
>104JA Pangiprobe
CTCACGCCATATCCGCCATTCTGACCTTTTTCCAGTG-AAGAATATTTGGGTTAGGGTCCC
>106JA Pangiprobe

CTCACGCCATATCCGCCATTCTGACCTTTTTCCAGTG-AAGAATATTTGGGTTAGGGTCCC
>107NBT Pangiprobe
CTCACGCCATATCCGCCATTCTGACCTTTTTCCAGTG-AAGAATATTTGGGTTAGGGTCCC
>125NAT Pangiprobe
CTCACGCCATATCCGCCATTCTGACCTTTTTCCAGTG-AAGAATATTTGGGTTAGGGTCCC
>179NAT Pangiprobe
CTCACGCCATATCCGCCATTCTGACCTTTTTCCAGTG-AAGAATATTTGGGTTAGGGTCCC
>180NAT Pangiprobe
CTCACGCCATATCCGCCATTCTGACCTTTTTCCAGTG-AAGAATATTTGGGTTAGGGTCCC
>182NAT Pangiprobe
CTCACGCCATATCCGCCATTCTGACCTTTTTCCAGTG-AAGAATATTTGGGTTAGGGTCCC
>183NAT Pangiprobe
CTCACGCCATATCCGCCATTCTGACCTTTTTCCAGTG-AAGAATATTTGGGTTAGGGTCCC
>264NAT Pangiprobe (Labelled as *P. ginseng* but not amplified by *P. ginseng* probe)
CTCACCCCATATCCGCCATTCTGACCTTTTTCCAGTG-AAGAATATTTGGGTTAGGGTCCC
>265NAT Pangiprobe
CTCACGCCATATCCGCCATTCTGACCTTTTTCCAGTG-AAGAATATTTGGGTTAGGGTCCC
>280NAT Pangiprobe
CTCACGCCATATCCGCCATTCTGACCTTTTTCCAGTG-AAGAATATTTGGGTTAGGGTCCC
>342NAT Pangiprobe
CTCACGCCATATCCGCCATTCTGACCTTTTTCCAGTG-AAGAATATTTGGGTTAGGGTCCC
>350NAT Pangiprobe (Labelled as *P. ginseng* but not amplified by *P. ginseng* probe)
CTCACCCCATATCCGCCATTCTGACCTTTTTCCAGTG-AAGAATATTTGGGTTAGGGTCCC
>3IND Pangiprobe
CTCACGCCATATCCGCCATTCTGACCTTTTTCCAGTG-AAGAATATTTGGGTTAGGGTCCC
>487NAT Pangiprobe
CTCACGCCATATCCGCCATTCTGACCTTTTTCCAGTG-AAGAATATTTGGGTTAGGGTCCC
>488NAT Pangiprobe
CTCACGCCATATCCGCCATTCTGACCTTTTTCCAGTG-AAGAATATTTGGGTTAGGGTCCC
>74NAT Pangiprobe
CTCACGCCATATCCGCCATTCTGACCTTTTTCCAGTG-AAGAATATTTGGGTTAGGGTCCC
>86NW Pangiprobe
CTCACGCCATATCCGCCATTCTGACCTTTTTCCAGTG-AAGAATATTTGGGTTAGGGTCCC
>BRM688 Pangiprobe
CTCACGCCATATCCGCCATTCTGACCTTTTTCCAGTG-AAGAATATTTGGGTTAGGGTCCC
>106NBT Pangiprobe
CTCACGCCATATCCGCCATTCTGACCTTTTTCCAGTG-AAGAATATTTGGGTTAGGGTCCC
>184NAT Pangiprobe
CTCACGCCATATCCGCCATTCTGACCTTTTTCCAGTG-AAGAATATTTGGGTTAGGGTCCC
>347NAT Pangiprobe
CTCACGCCATATCCGCCATTCTGACCTTTTTCCAGTG-AAGAATATTTGGGTTAGGGTCCC
>351NAT Pangiprobe
CTCACGCCATATCCGCCATTCTGACCTTTTTCCAGTG-AAGAATATTTGGGTTAGGGTCCC

>368NAT Pangiprobe
CTCACGCCATATCCGCCATTCTGACCTTTTTCCAGTG-AAGAATATTTGGGTTAGGGTCCC
>NAT-Nov-2020 Pangiprobe
CTCACGCCATATCCGCCATTCTGACCTTTTTCCAGTG-AAGAATATTTGGGTTAGGGTCCC
>BRM689 Pangiprobe
CTCACGCCATATCCGCCATTCTGACCTTTTTCCAGTG-AAGAATATTTGGGTTAGGGTCCC
>JFN1 Pangiprobe
CTCACGCCATATCCGCCATTCTGACCTTTTTCCAGTG-AAGAATATTTGGGTTAGGGTCCC

Table S9. Amplicon sequences obtained from *Panax quinquefolius* commercial products

>249NAT _Panquprobe
GGTTGCTTTCTGTCCATATAATGCATACAGCTCTAGTTGCCGGT
>OAC1570 _Panquprobe
GGTTGCTTTCTGTCCATATAATGCATACAGCTCTAGTTGCCGGT
>348NAT _Panquprobe
GGTTGCTTTCTGTCCATATAATGCATACAGCTCTAGTTGCCGGT
>PQ21 _Panquprobe
GGTTGCTTTCTGTCCATATAATGCATACAGCTCTAGTTGCCGGT
>PQ23 _Panquprobe
GGTTGCTTTCTGTCCATATAATGCATACAGCTCTAGTTGCCGGT
>339NAT _Panquprobe
GGTTGCTTTCTGTCCATATAATGCATACAGCTCTAGTTGCCGGT
>351NAT _Panquprobe
GGTTGCTTTCTGTCCATATAATGCATACAGCTCTAGTTGCCGGT
>406NW _Panquprobe
GGTTGCTTTCTGTCCATATAATGCATACAGCTCTAGTTGCCGGT
>415NAT _Panquprobe
GGTTGCTTTCTGTCCATATAATGCATACAGCTCTAGTTGCCGGT
>OAC26065 _Panquprobe
GGTTGCTTTCTGTCCATATAATGCATACAGCTCTAGTTGCCGGT
>PQ25 _Panquprobe
GGTTGCTTTCTGTCCATATAATNCATACAGCTCTAGTTGCCGGT
>1084NW _Panquprobe
GGTTGCTTTCTGTCCATATAATGCATACAGCTCTAGT-GCCGGT
>OAC57071 _Panquprobe
GGTTGCTTTCTGTCCATATAATGCATACAGCTCTAGT-GCCGGT
>OAC74790 _Panquprobe
GGTTGCTTTCTGTCCATATAATGCATACAGCTCTAGT-GCCGGT
>112NBT _Panquprobe

GGTTGCTTTCTGTCCATATAATGCATACAGCTCTAGT-GCCGGT
>TNP1 _Panquprobe
GGTTGCTTTCTGTCCATATAATGCATACAGCTCTAGT-GCCGGT
>BRM705 _Panquprobe
GGTTGCTTTCTGTCCATATAATGCATACAGCTCTAGT-GCCGGT
>BRM871 _Panquprobe
GGTTGCTTTCTGTCCATATAATGCATACAGCTCTAGT-GCCGGT
>BRM903 _Panquprobe
GGTTGCTTTCTGTCCATATAATGCATACAGCTCTAGT-GCCGGT
>OAC24225 _Panquprobe
GGTTGCTTTCTGTCCATATAATGCATACAGCTCTAGT-GCCGGT
>PQ24 _Panquprobe
GGTTGCTTTCTGTCCATATAATGCATACAGCTCTAGT-GCCGGT
>PQ27 _Panquprobe
GGTTGCTTTCTGTCCATATAATGCATACAGCTCTAGT-GCCGGT
>PQ30 _Panquprobe
GGTTGCTTTCTGTCCATATAATGCATACAGCTCTAGT-GCCGGT
>OAC17943 _Panquprobe
GGTTGCTTTCTGTCCATATAATGCATACAGCTCTAGT-GCCGGT
>OAC92518 _Panquprobe
GGTTGCTTTCTGTCCATATAATGCATACAGCTCTAGT-GCCGGT
>PQ29 _Panquprobe
GGTTGCTTTCTGTCCATATAATGCATACAGCTCTAGT-GCCGGT
>PQ22 _Panquprobe
GGTTGCTTTCTGTCCATATAATGCATACAGCTCTAGT-GCCGGT
>OAC42034 _Panquprobe
GGTTGCTTTCTGTCCATATAATG-ATACAGCTCTAGT-GCCGGT
>14NAT _Panquprobe
GGTTGCTTTCTGTCCATATAATGCATACAGCTCTAGT-GCCGGT
>347NAT _Panquprobe
GGTTGCTTTCTGTCCATATAATGCATACAGCTCTAGT-GCCGGT
>BRM803 _Panquprobe
GGTTGCTTTCTGTCCATATAATGCATACAGCTCTAGT-GCCGGT
>PQ26 _Panquprobe
GGTTGCTTTCTGTCCATATAATGCATACAGCTCTAGT-GCCGGT
>OAC56612 _Panquprobe
GGTTGCTTTCTGTCCATATAATGCATACAGCTCTAGT-GCCGGT
>7PR _Panquprobe
GGTTGCTTTCTGTCCATATAATGCATACAGCTCTAGT-GCCGGT
>OAC22568 _Panquprobe
GGTTGCTTTCTGTCCATATAATGCATACAGCTCTAGT-GCCGGT
>8PR _Panquprobe
GGTTGCTTTCTGTCCATATAATGCATACAGCTCTAGT-GCCGGT

>PQ28 _Panquprobe
---NTCTTTCTGTCCATATAATGCATACAGCTCTAGT-GCCGGT
>24PR _Panquprobe
GGTTGCTTTCTGTCCATATAATGCATACAGCTCTAGT-GCCGGT
>17NAT _Panquprobe
GGTTGCTTTCTGTCCATATAATG-ATACAGCTCTAGTTGCCGGT
>OAC56582 _Panquprobe
GGTTGCTTTCTGTCCATATAATGCATACAGCTCTAGT-GCCGGT

Panax ginseng amplicon:AAA**GTCTTGGATAGCCGCGATAAA**TCCAAATCTGGCTCACGCCATATCCGCCATTCTGACCTTTTTCCAGTGA**AAGAATATTTGGGTTAGGGTCCC**CCA

Panax quinquefolius: AAA**GTCTTGGATAGCCGCGATAAA**TCCAAATCTGGCTCACCCCATATCCGCCATTCTGACCTTTTTCCAGTGA**AAGAATATTTGGGTTAGGGTCCC**CCA

Primer 1 **Probe** **Primer 2**

Fig S1. ***Panax ginseng*** **amplicon sequence showing variability with** ***Panax quinquefolius***

Panax quinquefolius amplicon: CGG**CCGGTTGCTTTCTGTCCATATAATG**CATACAGCTCTAGTTGCCGGTTGGGCGGGTTCGATGGCTCTATATGAATTAGCAGTTT**TTGATCCCTCTGACCCTGTT**CTT

Panax ginseng: CGG**CCGGTTGCTTTCTGTCCATATAATG**CATACAGCTCTAGTTGCTGGTTGGGCGGGTTCGATGGCTCTATATGAATTAGCAGTTT**TTGATCCCTCTGACCCTGTT**CTT

Primer 1 **Probe** **Primer 2**

Fig S2. ***Panax quinquefolius*** **amplicon sequence showing variability with** ***Panax ginseng***
